# Genomic characterization of efflux pumps and putative small multidrug resistance (SMR)-like transporters in disinfectant-tolerant coagulase-negative staphylococci from a Vietnamese hospital

**DOI:** 10.1128/spectrum.01294-26

**Published:** 2026-09-09

**Authors:** Dao Hai Hung, Le Phung Hien, Pham Kieu Oanh, Ngo Thi Thanh Huyen, Ha Thi Viet Bui

**Affiliations:** 1 Center for Life Sciences Research (CELIFE), Faculty of Biology, VNU University of Science 597279 , Hanoi, Vietnam; 2 College of Science and Engineering, Flinders University 1065, Adelaide, South Australia, Australia; Universidad Nacional Autonoma de Mexico - Campus Morelos, Cuernavaca, Mexico

**Keywords:** efflux-mediated disinfectant tolerance, coagulase-negative staphylococci, *Staphylococcus nepalensis*, *Staphylococcus haemolyticus*

## Abstract

Coagulase-negative staphylococci (CoNS) are common inhabitants of healthcare environments and can serve as reservoirs of antimicrobial resistance determinants. However, the relationship among disinfectant tolerance, efflux-associated phenotypes, and multidrug resistance in environmental CoNS remains incompletely defined. Here, 51 staphylococcal isolates were recovered from high-touch hospital surfaces in Hanoi, Vietnam, and 14 isolates showed elevated MICs to at least one disinfectant, including benzalkonium chloride, chlorhexidine gluconate, or hydrogen peroxide, relative to *Staphylococcus aureus* ATCC 25923. These isolates also displayed multidrug-resistant phenotypes. Moreover, ethidium bromide accumulation assays revealed a clear efflux-associated phenotype, and representative isolates exhibited biofilm-forming ability. Whole-genome sequencing of three representative isolates reclassified SDH.B1 and SDH.B3 as *Staphylococcus nepalensis*—a CoNS species rarely documented in hospital AMR surveillance—and confirmed SDH.B7 as *Staphylococcus haemolyticus*. To our knowledge, this is among the first reports of *S. nepalensis* carrying *mecA* together with the disinfectant efflux gene *qacG*, and a set of putative small multidrug resistance (SMR)-like transporters in a hospital environment from Southeast Asia. In addition, six putative SMR-like proteins were identified. Phylogenetic and structural analyses suggested relatedness to YkkC/YkkD-like systems, although their transport activity remains to be experimentally verified. These findings identify environmental CoNS as reservoirs of disinfectant tolerance and multidrug resistance in hospital settings and provide a basis for future functional investigation of putative SMR-like transporters.

**IMPORTANCE:**

Hospital environmental CoNS are often dismissed as low-priority contaminants, yet they may persist under disinfectant pressure and carry clinically relevant resistance determinants. Here, disinfectant-tolerant CoNS recovered from a Vietnamese hospital combined multidrug resistance, efflux-associated phenotypes, and biofilm-related traits. Genome analysis further revealed known *qac* genes and six putative SMR-like proteins that merit functional testing. These findings support inclusion of environmental CoNS in infection-prevention surveillance and disinfectant stewardship programs.

## INTRODUCTION

Antimicrobial resistance is driven by multiple mechanisms, including active efflux, target modification, enzymatic inactivation, and biofilm formation. Together, these traits reduce the efficacy of antibiotics and disinfectants and complicate infection prevention in both clinical and environmental settings. The global burden of antimicrobial resistance (AMR) is substantial, with an estimated 1.27 million deaths directly attributable to bacterial resistance in 2019 (1). In Vietnam, multidrug-resistant bacteria are increasingly reported in hospitals and community settings (2, 3).

Among hospital-associated bacteria, staphylococci are especially important because they can persist on abiotic surfaces, colonize patients and healthcare workers, and act as reservoirs of mobile resistance determinants. Although *Staphylococcus aureus* remains the best-studied species, coagulase-negative Staphylococci (CoNS) are increasingly recognized as opportunistic pathogens and resilient environmental survivors. Within this group, species such as *S. epidermidis* and *S. haemolyticus* have been studied for their resistance potential, but other CoNS—including *S. nepalensis*, a species almost absent from hospital AMR literature—remain poorly characterized in healthcare contexts despite their capacity to acquire mobile resistance elements (4). Their persistence is facilitated by tolerance to environmental stress, biofilm formation, and the acquisition of resistance genes. These features raise concern that CoNS may survive routine cleaning and disinfection and contribute to continued contamination of healthcare environments. Disinfectants such as benzalkonium chloride (BKC) and chlorhexidine gluconate (CHG) are widely used in healthcare settings for surface decontamination, hand hygiene, and device-associated infection prevention (5–9).

Reduced susceptibility to biocidal agents in staphylococci is most commonly mediated by active efflux, with *qac*-encoded systems representing the best-characterized determinants. QacA and QacB belong to the major facilitator superfamily (MFS), while QacC is a member of the small multidrug resistance (SMR) family (10–13). SMR transporters are of particular interest because of their compact size, conserved proton-motive-force-dependent mechanism, and capacity to effectively extrude quaternary ammonium compounds. Efflux-mediated tolerance is further compounded by biofilm formation. Within a biofilm, bacteria are encased in an extracellular matrix that limits disinfectant diffusion, while high cell-surface hydrophobicity promotes adhesion to abiotic surfaces and facilitates biofilm establishment. Together, these traits confer a layered survival advantage that operates at both the molecular and community level. Yet despite their clear relevance to hospital hygiene, the full repertoire of SMR-like proteins in environmental CoNS has not been examined, and the degree to which uncharacterized variants contribute to persistence under disinfectant pressure remains unknown. This gap is especially consequential in healthcare settings, where ongoing antibiotic and biocide use creates sustained selective pressure. Studies that couple phenotypic susceptibility testing with whole-genome sequencing are therefore needed to capture the true diversity of efflux determinants and to clarify how efflux activity, multidrug resistance, and biofilm formation interact in hospital-associated CoNS.

In this study, we investigated staphylococcal isolates recovered from high-touch surfaces in a Vietnamese hospital to assess the burden of disinfectant tolerance and antibiotic resistance among environmental strains. We combined phenotypic susceptibility testing, ethidium bromide accumulation assays, biofilm-related characterization, and whole-genome sequencing of representative multidrug-resistant isolates to define their resistome and efflux pump repertoire. Emphasis was placed on identifying previously uncharacterized SMR-like transporters that may contribute to reduced disinfectant susceptibility. By characterizing both known and putative resistance determinants in hospital-associated CoNS—including species such as *S. nepalensis* that are largely overlooked in routine surveillance—this study provides insight into the potential role of staphylococci in the persistence and dissemination of multidrug-resistant populations in healthcare settings.

## MATERIALS AND METHODS

### Bacterial isolation and preliminary identification

Environmental samples were collected from high-touch surfaces at a hospital in Hanoi, Vietnam, between July 2024 and January 2025 using sterile medical cotton swabs (MediSafe). Sampling sites included frequently contacted areas such as emergency rooms, restrooms, hallways, elevators, and handwashing stations. Three independent samples were collected from each location. Swabs were inoculated onto Mannitol Salt Agar and incubated at 37°C for 24 to 72 h. Presumptive staphylococcal isolates were screened by colony morphology, Gram staining, and catalase testing and were retained for further phenotypic characterization.

### Species identification by MALDI-TOF MS

Colonies with elevated disinfectant MICs were identified by matrix-assisted laser desorption ionization–time of flight mass spectrometry using the MALDI-TOF VITEK MS system (bioMérieux, France) according to the manufacturer’s instructions. MALDI-TOF served as the initial species identification method before selection of representative isolates for whole-genome sequencing (13).

### Determination of disinfectant minimum inhibitory concentrations

Minimum inhibitory concentrations (MICs) of benzalkonium chloride (BKC), chlorhexidine gluconate (CHG), and hydrogen peroxide (H₂O₂) were determined by broth microdilution in accordance with standard laboratory practice based on CLSI-guided susceptibility testing principles. Bacterial cultures were grown to mid-log phase and adjusted to an optical density of approximately OD_600_ = 0.6 before dilution to a final inoculum of approximately 5 × 10⁵ CFU/mL in Mueller-Hinton broth. Aliquots were dispensed into 96-well microplates containing serial dilutions of each disinfectant and incubated at 37°C for 24–48 h. After incubation, resazurin was added as a viability indicator, and the MIC was defined as the lowest concentration that prevented a visible color change from blue to pink. *S. aureus* ATCC 25923 was used as the reference strain. The experiment was repeated three times.

### Antibiotic susceptibility testing

Antibiotic susceptibility profiles were determined using the VITEK 2 Compact system (bioMérieux, France). Fresh bacterial colonies were suspended in sterile 0.9% NaCl and adjusted to a 0.5 McFarland standard prior to loading into the system. Automated incubation and optical monitoring were performed according to the manufacturer’s recommendations, and isolates were categorized as susceptible, intermediate, or resistant on the basis of CLSI interpretive criteria. For antibiotics that could not be evaluated by the VITEK 2 system due to the lack of species-specific validated interpretive criteria, susceptibility was instead assessed by the Kirby–Bauer disk diffusion method (CLSI M02), with zone diameters interpreted according to CLSI M100 criteria. Individual antibiotics were grouped into antibiotic classes to provide a more holistic assessment of resistance patterns, and isolates were classified as multidrug-resistant (MDR) according to Magiorakos et al (14), i.e., acquired non-susceptibility to at least one agent in three or more antimicrobial categories.

### Ethidium bromide accumulation assay

To assess efflux-associated activity, intracellular accumulation of ethidium bromide (EtBr) was measured fluorometrically, adapted from Blair et al., with some modifications (15). Bacterial isolates were grown to mid-log phase in LB medium, harvested by centrifugation, and washed three times with phosphate-buffered saline (PBS). Cell suspensions were adjusted to OD_600_ = 1.0. For each reaction, 100 µL of bacterial suspension was added to a fluorescence-compatible microplate. To establish the baseline autofluorescence of the bacterial cells, fluorescence was monitored at 37°C for the first 5 min. EtBr and glucose were added to the wells to achieve final concentrations of 20 µM EtBr and 200 mM glucose, an energy source to drive active efflux (with or without a final concentration of 25 µg/mL nicardipine to block active efflux). The use of nicardipine as a bacterial efflux pump inhibitor (EPI) was chosen from previous studies (16). At this concentration, nicardipine did not inhibit bacterial growth and did not alter fluorescence in cell-free control wells. Fluorescence monitoring was then continued for an additional 60 min at 1-min intervals (total assay time of 66 min) using excitation and emission wavelengths of 555 and 615 nm on a Varioskan ALF Microplate Reader (Thermo Fisher Scientific). To obtain the net intracellular accumulation signal, the raw fluorescence values recorded after the addition of the treatments were corrected by subtracting the background fluorescence of the cell-free controls (i.e., the dye-only and dye + nicardipine only wells) at each corresponding time point. Reduced net fluorescence accumulation over time in the test isolates relative to the reference strains was interpreted as active efflux-associated activity, which was expected to yield elevated intracellular EtBr accumulation upon the addition of the efflux inhibitor nicardipine.

### Biofilm formation assay

Biofilm formation was assessed using the microtiter plate assay described by Stepanović et al., with minor modifications (17). Bacterial isolates were first cultured in Tryptic Soy Broth (TSB) to approximately OD_600_ = 0.6 and then diluted to 0.1. For each well, 100 µL of TSB was combined with 10 µL of the diluted culture in a sterile 96-well plate. Plates were briefly shaken at 120 rpm for 10 min and then incubated statically at 37°C for 48 h. Following incubation, planktonic cells were removed and wells were gently rinsed to eliminate non-adherent cells. Plates were dried and fixed at 60°C for 1 h, stained with 0.1% safranin O for 30 min, and washed to remove excess dye. Bound stain was solubilized with 33% acetic acid, and absorbance was measured at 485 nm.

### Microbial adhesion to hydrocarbons (MATH) assay

Cell-surface hydrophobicity was evaluated using the microbial adhesion to hydrocarbons assay, adapted from Salas-Tovar et al., with some modifications (18). Bacterial cultures were grown to late log phase, washed, and resuspended in PBS to OD_600_ = 1.0. A 5 mL aliquot of the bacterial suspension was mixed with 1 mL of n-octane and vortexed vigorously for 2 min. The mixture was allowed to stand undisturbed for 15 min to permit phase separation. The OD_600_ of the aqueous phase was then measured, and hydrophobicity was calculated as the percentage decrease in optical density relative to the initial suspension. Higher adhesion values were interpreted as indicating greater cell-surface hydrophobicity.

### Selection of representative isolates for whole-genome sequencing

Three isolates—SDH.B1, SDH.B3, and SDH.B7—were selected for whole-genome sequencing based on a combination of criteria: each represented a distinct species by MALDI-TOF MS, carried the highest MICs to some tested biocides, and met the multidrug resistance criterion. All three also showed near-baseline EtBr fluorescence, indicating pronounced efflux-associated activity. Furthermore, these isolates were chosen to encompass a diverse spectrum of biofilm-forming capabilities and cell-surface hydrophobicity profiles—ranging from the low hydrophobicity of SDH.B1 to the strong, statistically significant biofilm formation and high hydrophobicity of SDH.B7. Together, these complementary criteria made the three isolates representative models for investigating the genetic basis of the efflux-driven, disinfectant-tolerant phenotypes observed across the broader collection.

Genomic DNA was extracted using a phenol-chloroform-isoamyl alcohol protocol adapted from published methods (19–21). Cells were harvested by centrifugation, resuspended in Tris-HCl buffer, treated with lysozyme, and digested with Proteinase K and RNase A. DNA was then purified by phenol-chloroform-isoamyl alcohol extraction, precipitated with ethanol and sodium acetate, washed with 70% ethanol, and air-dried. Whole-genome sequencing was performed by Azenta company using 150-bp paired-end reads on the Illumina NovaSeq 6000 instrument according to manufacturer’s instructions (Illumina, San Diego, CA, USA).

### Genome assembly and annotation

Raw sequencing reads were subjected to quality control using FastQC (v0.12.1) and processed for trimming and filtering using BBMap (v35.85). High-quality reads were assembled *de novo* with Unicycler (v0.5.1) using multiple k-mer sizes (22). Assembly graphs were visualized using Bandage, and genome annotation was performed with Bakta (v1.11) (23, 24). These procedures generated draft genomes suitable for downstream comparative genomic and resistome analyses.

### Phylogenetic, resistome, and comparative genomic analyses

Phylogenetic relationships were inferred from conserved housekeeping genes (listed in Supplemental data) using IQ-TREE (v2.2.0) with 10,000 bootstrap replicates, and phylogenetic trees were visualized using iTOL v6 (25, 26). Average nucleotide identity based on the OrthoANIu algorithm was used to refine species assignments of the sequenced isolates (27). AMR genes and efflux-associated determinants were identified using AMRFinderPlus (v4.0.22) (28). Multilocus sequence typing was performed using the CGE platform where applicable (29). Staphylococcal cassette chromosome *mec* (SCC*mec*) elements were characterized using SCCmecFinder v1.2, and plasmid reconstruction and typing were performed using MOB-suite v3.1.9 to assess the dissemination potential of resistance genes (30, 31). The Circos plot was generated using custom-developed code based on the design style of the Circos software (32). Comparative analyses were used to examine the resistome composition and to identify genes potentially associated with disinfectant tolerance and multidrug resistance.

### Identification and characterization of putative SMR-like proteins

To characterize newly identified small multidrug resistance (SMR)-like proteins, predicted amino acid sequences were analyzed for physicochemical properties using ExPASy ProtParam (33). Multiple sequence alignments were generated with MUSCLE (v5) and visualized in UGENE. Three-dimensional heterodimeric structures were predicted using AlphaFold 2 (AF2), specifically employing the alphafold2_multimer_v3 model with no template (34). To maximize prediction accuracy, the computational pipeline was executed with 24 recycling iterations. MSAs were generated with MMseqs2 and HHsearch, and five models were predicted per complex, from which the top-ranked model was selected for downstream analysis. Model reliability was assessed using AF2 confidence metrics, including per-residue pLDDT, PAE, pTM, and ipTM. Per-residue pLDDT mapped onto 3D structures, and PAE plots are provided in the Supplemental data. AF2-predicted uSMR heterodimers were also superimposed onto the reference YkkC/YkkD complex using the align command in PyMOL (Schrödinger, LLC). Quantitative structural comparisons were performed using the MM-align algorithm via the Zhang Lab web server to calculate the Template Modeling score (TM-score) and root-mean-square deviation (RMSD) (35). For all pairwise structural comparisons, length normalization for both TM-score and RMSD was based on the longer protein within each compared pair. Pairwise sequence identity was calculated via the Zhang Lab web server as the ratio of identical residues to the total number of aligned residues.

### Statistical analysis

Quantitative data from EtBr accumulation, biofilm formation, and MATH assays are summarized as mean ± standard deviation. The EtBr quantification experiment was repeated three times, while MATH and biofilm formation assessments were repeated six times. Robust statistical methods based on trimmed means were performed using R Statistical Software v4.6.1 (via the WRS2 package). Specifically, differences between groups for biofilm formation and MATH assays were assessed using a one-way robust ANOVA (t1way), followed by post hoc comparisons with Holm’s correction. For the EtBr accumulation assay, a two-way robust ANOVA (t2way) coupled with the mcp2a post hoc test was applied to compare the area under the curve (AUC) before and after nicardipine supplementation, as well as to determine the effect size of the EPI. A *P* value < 0.05 was considered statistically significant.

## RESULTS

### Recovery of disinfectant-tolerant staphylococci from hospital surfaces

A total of 51 staphylococcal isolates were recovered from high-touch surfaces in the hospital environment. Among these, 14 isolates (27%) exhibited elevated MIC to at least one disinfectant relative to the reference strain *S. aureus* ATCC 25923 (Table 1).

**TABLE 1:** MALDI-TOF MS identification of 14 hospital-derived staphylococcal isolates and their minimum inhibitory concentrations (MICs) to benzalkonium chloride (BKC), chlorhexidine gluconate (CHG), and hydrogen peroxide (H_2_O_2_)

Sample	MS identification	MIC (µg/mL)		
		BKC	CHG	H_2_O_2_
SDH.B1	*S. ureilyticus*	16	0.2	64
SDH.B2	*S. equorum*	16	0.2	32
SDH.B3	*S. equorum*	16	0.2	32
SDH.B5	*S. haemolyticus*	8	0.2	32
SDH.B7	*S. haemolyticus*	8	0.8	64
SDH.B8	*S. haemolyticus*	8	0.8	64
SDH.B10	*S. haemolyticus*	8	0.8	64
SDH.B15	*S. ureilyticus*	8	0.4	64
SDH.B30	*S. cohnii*	8	0.8	64
SDH.B32	*S. cohnii*	8	0.2	64
SDH.B34	*S. cohnii*	8	0.2	64
SDH.B36	*S. cohnii*	8	0.8	64
SDH.B39	*S. cohnii*	8	0.4	64
SDH.B51	*S. cohnii*	8	0.2	64
*S. aureus* ATCC 25923		2	0.4	32

Reduced susceptibility to BKC was common, with MICs clustering at 8–16 µg/mL, corresponding to approximately 4- to 8-fold higher values than those of the reference strain. Hydrogen peroxide MICs ranged from 32 to 64 µg/mL, whereas CHG MICs were more heterogeneous, with a subset of isolates showing a 2-fold increase relative to the reference. Initial MALDI-TOF MS identification assigned all 14 isolates to CoNS, including *S. cohnii*, *S. haemolyticus*, *S. equorum*, and *S. ureilyticus*.

### Antibiotic susceptibility profiles revealed widespread multidrug resistance

The disinfectant-tolerant isolates also showed broad resistance to multiple antibiotic classes. All 14 isolates were resistant to β-lactams and macrolides, consistent with a methicillin-resistant phenotype. Resistance to lincosamides and tetracyclines was also common, while resistance to aminoglycosides, trimethoprim, and fluoroquinolones was comparatively less frequent, occurring in a minority of isolates. In contrast, all isolates remained susceptible to glycopeptides (Table 2). Collectively, these findings indicate that reduced disinfectant susceptibility in the hospital-derived CoNS was commonly accompanied by multidrug resistance.

**TABLE 2:** Antimicrobial susceptibility profiles of the studied isolates^*a*^

Sample	β-Lactams	Macrolides	Lincosamides	Tetracyclines	Aminoglycosides	Glycopeptides	Fluoroquinolones	Trimethoprim
SDH.B1	**R**	**R**	**R**	**R**	S	S	**R**	**R**
SDH.B2	**R**	**R**	**R**	**R**	**R**	S	**R**	*–*
SDH.B3	**R**	**R**	**R**	S	**R**	S	**R**	*–*
SDH.B5	**R**	**R**	**R**	**R**	S	S	S	S
SDH.B7	**R**	**R**	**R**	**R**	**R**	S	S	S
SDH.B8	**R**	**R**	**R**	**R**	S	S	S	S
SDH.B10	**R**	**R**	**R**	**R**	**R**	S	S	S
SDH.B15	**R**	**R**	**R**	*–*	S	S	S	*–*
SDH.B30	**R**	**R**	*–*	**R**	S	S	S	*–*
SDH.B32	**R**	**R**	**R**	**R**	S	S	S	*–*
SDH.B34	**R**	**R**	*–*	**R**	S	S	S	*–*
SDH.B36	**R**	**R**	**R**	**R**	S	S	S	*–*
SDH.B39	**R**	**R**	*–*	**R**	S	S	S	*–*
SDH.B51	**R**	**R**	*–*	**R**	S	S	S	*–*

^*a*^ **R**, intermediate or resistant to at least one antibiotic within the class; S, susceptible to antibiotics tested within the class; –, not determined.

### Ethidium bromide accumulation assays suggested strong efflux-associated activity

**Fig 1:**
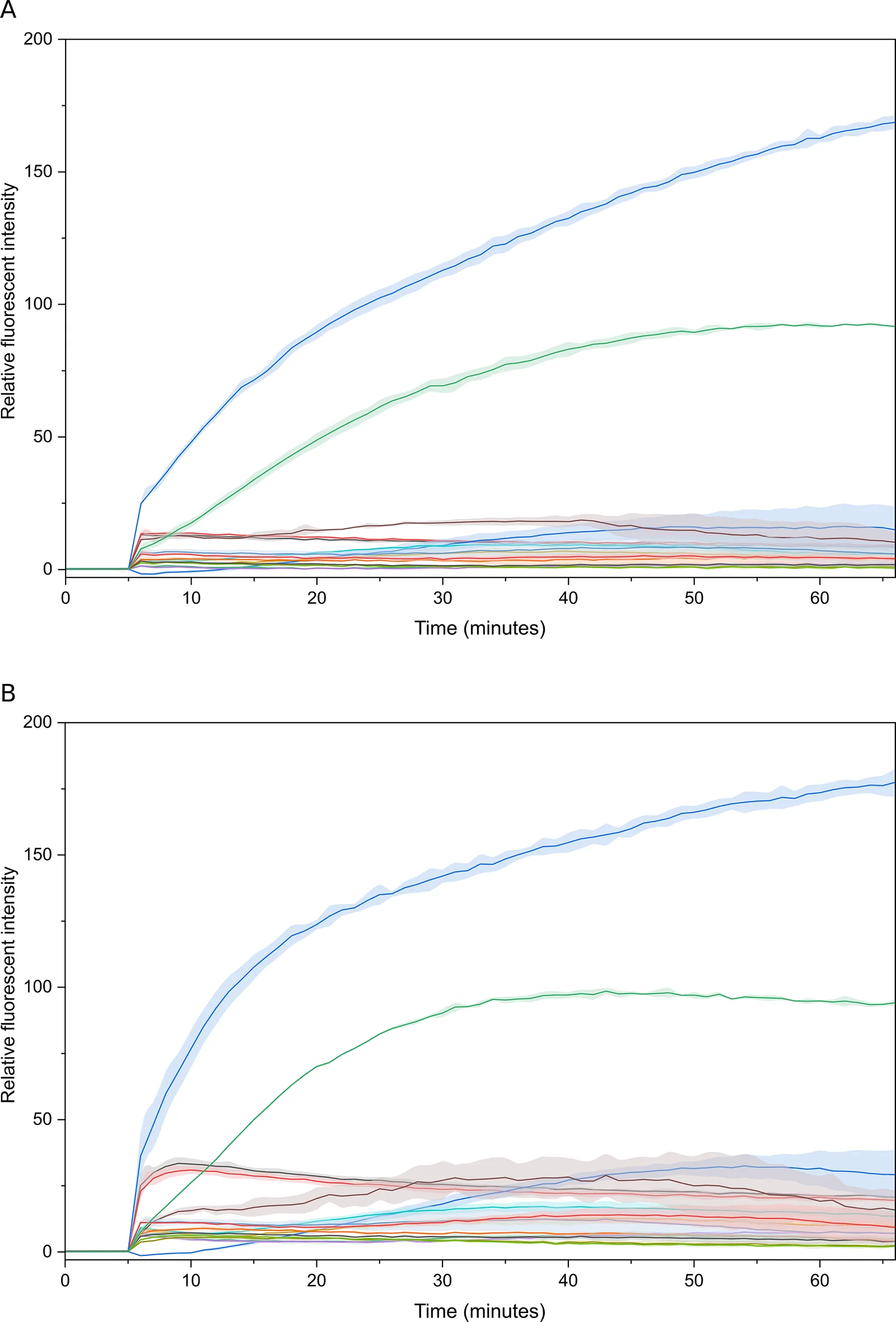
Real-time kinetics of net intracellular ethidium bromide (EtBr) accumulation in *Staphylococcus* isolates. (**A**) EtBr accumulation kinetics in the absence of an efflux pump inhibitor. (**B**) EtBr accumulation kinetics in the presence of an efflux pump inhibitor. The initial 5 min depict the pre-addition baseline monitoring of bacterial autofluorescence prior to dye/inhibitor introduction. Values reflect net fluorescence intensity obtained after subtracting cell-free background controls at each corresponding time point. Error ribbons denote standard deviations from three replicates. Legend: One color band corresponds to one isolate.

**Fig 2:**
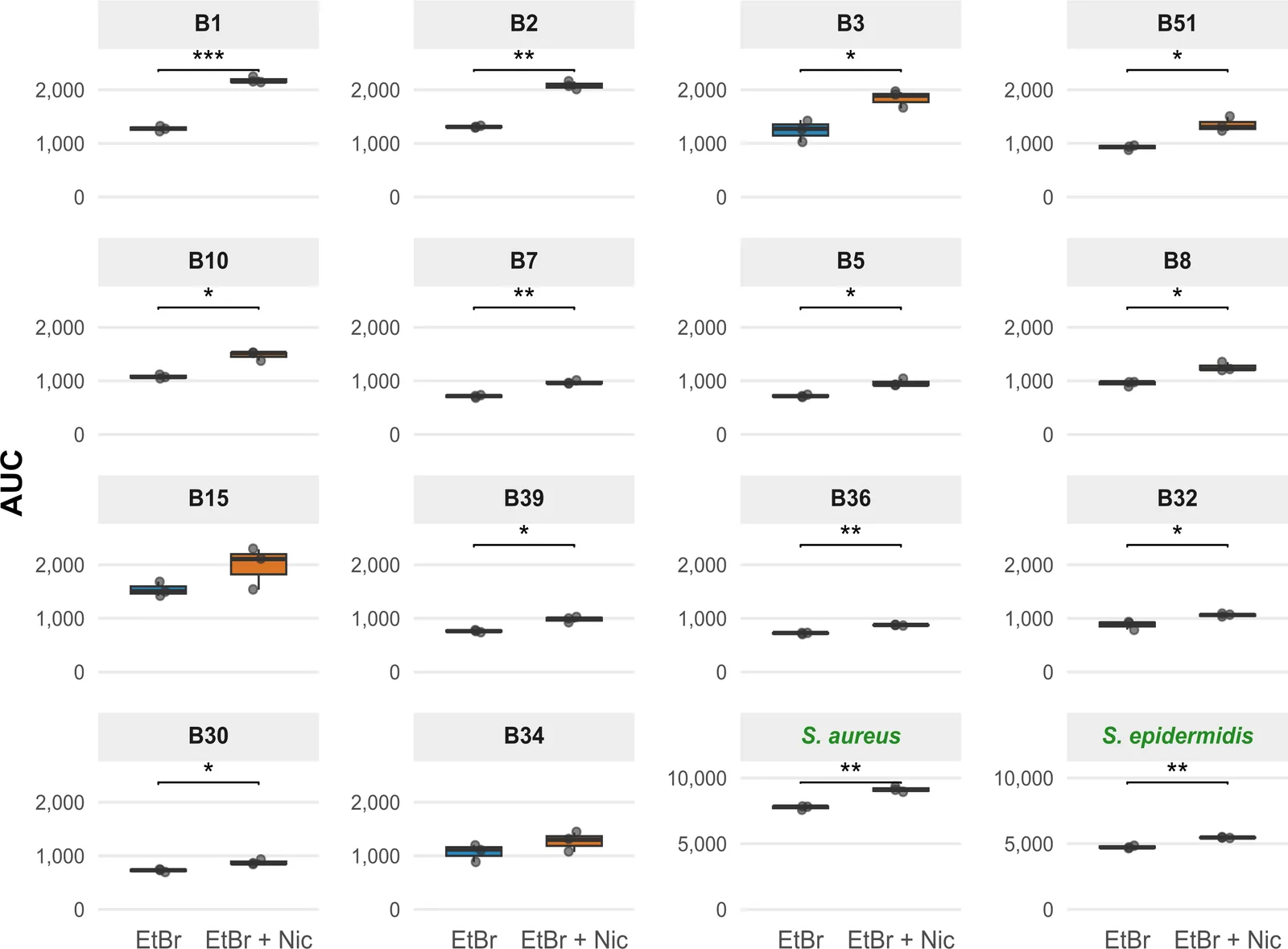
Quantitative comparison of EtBr accumulation with and without Nicardipine (Nic) in *Staphylococcus* isolates. AUC was calculated from the accumulation kinetics shown in Fig. 1 (*P < 0.05, **P < 0.01, ***P < 0.001).

To explore whether reduced disinfectant susceptibility was associated with active efflux, intracellular EtBr accumulation was monitored over time in both the absence and presence of the EPI nicardipine. In the absence of EPI, the reference strains *S. aureus* ATCC 25923 and *S. epidermidis* ATCC 12228 showed progressive fluorescence accumulation, yielding substantially higher AUC values than the disinfectant-tolerant isolates, which instead maintained fluorescence close to baseline—a pattern consistent with active efflux limiting intracellular EtBr retention (Fig. 1A and Fig. 2). Upon addition of nicardipine, EtBr accumulation increased across all 16 strains, and this EPI-induced rise was statistically significant in 14 of them; only isolates B15 and B34 showed no significant difference (Fig. 1B and Fig. 2). The magnitude of this effect also varied between groups, with disinfectant-tolerant isolates showing up to a 1.7-fold increase in AUC, compared with a more modest 1.2-fold increase in the reference strains (Supplemental data). These kinetic and statistical findings point to efflux as a major contributor to disinfectant tolerance in those isolates.

### Representative multidrug-resistant isolates displayed enhanced biofilm formation and high surface hydrophobicity

**Fig 3:**
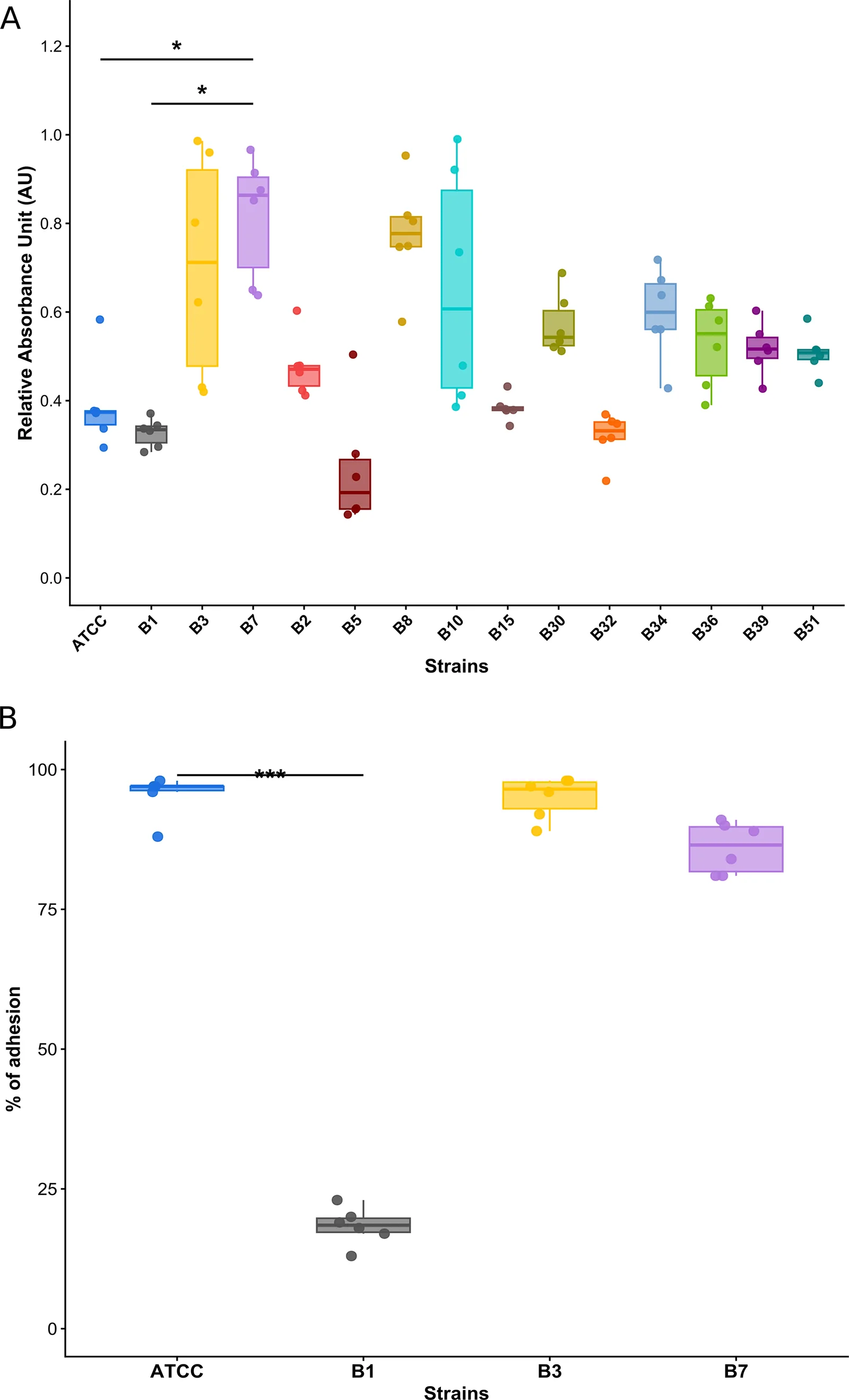
(**A**) Quantitative assessment of biofilm formation by 14 isolates compared with the reference strain *S. aureus* ATCC 25923. (**B**) Cell-surface hydrophobicity of representative strains SDH.B1, SDH.B3, and SDH.B7, alongside the *S. aureus* ATCC 25923, measured by the MATH assay. Asterisks indicate a significant difference between the indicated pair (*P < 0.05; ***P < 0.001).

Biofilm formation varied considerably among the disinfectant-tolerant isolates, with several strains producing more biofilm than the reference strain. Among the three representative multidrug-resistant isolates selected for further characterization, SDH.B3 and SDH.B7 exhibited biofilm-forming phenotypes, with median absorbance values of approximately 0.7 and 0.9 units, respectively. However, robust ANOVA with Holm’s correction revealed that only SDH.B7 produced significantly more biofilm than both the reference strain and SDH.B1 (Fig. 3A). Despite its high median absorbance, biofilm formation in SDH.B3 was highly variable and did not significantly exceed that of the reference strain. To determine whether this variation in biofilm-forming capacity was linked to differences in cell-surface properties, microbial adhesion to hydrocarbons was subsequently assessed. SDH.B3 and SDH.B7 both maintained high cell-surface hydrophobicity, comparable to the reference strain, with adhesion values above 85%. In contrast, SDH.B1 showed markedly lower hydrophobicity (<20%), significantly reduced compared to the reference strain (Fig. 3B).

### Whole-genome sequencing provided high-quality assemblies for three representative isolates

Based on their multidrug-resistant phenotypes and elevated disinfectant MICs, three representative isolates, SDH.B1, SDH.B3, and SDH.B7, were selected for *de novo* whole-genome sequencing. SDH.B1 and SDH.B3 assemblies are similar, with genomic sizes of around 2.95 Mb (2,948,700 and 2,946,593 bp), 32.87% GC, 62–69 contigs, and strong contiguity (N50 = 296,279 bp; L50 = 4) (Table 3). Annotations identify nearly 2,800 CDSs (2,803 and 2,798), 53 tRNAs each, and 6 and 5 rRNA genes, respectively. SDH.B7 has a smaller genome (2.54 Mb), a more fragmented assembly (120 contigs), and lower contiguity (N50 = 66,209 bp; L50 = 12), with 2,465 CDSs, 56 tRNAs, and 5 rRNAs. Overall GC content remains within the expected range for *Staphylococcus* (32.6%–32.9%) (36). The near-identical metrics for B1 and B3 reflect their identity as two isolates of the same *Staphylococcus nepalensis* strain. In contrast, B7’s smaller size and fragmentation point to either a genuine reduction in accessory content and/or unresolved repeats and plasmid regions at short-read resolution. These assemblies are sufficient for downstream analyses of gene content, antimicrobial resistance, and efflux determinants.

**TABLE 3:** Assembly statistics for the three representative multidrug-resistant *Staphylococcus* isolates sequenced in this study

Sample	Accession no.	Total genome size (bp)	No. of contigs	GC (%)	N50	L50	No. of CDS	No. of tRNA	No. of rRNA
SDH.B1	JBPUTG000000000	2,948,700	62	32.87	296,279	4	2,803	53	6
SDH.B3	JBPUTH000000000	2,946,593	69	32.87	296,279	4	2,798	53	5
SDH.B7	JBPUTI000000000	2,542,989	120	32.61	66,209	12	2,465	56	5

### Genome-based analyses refined species identification and sequence typing

**Fig 4:**
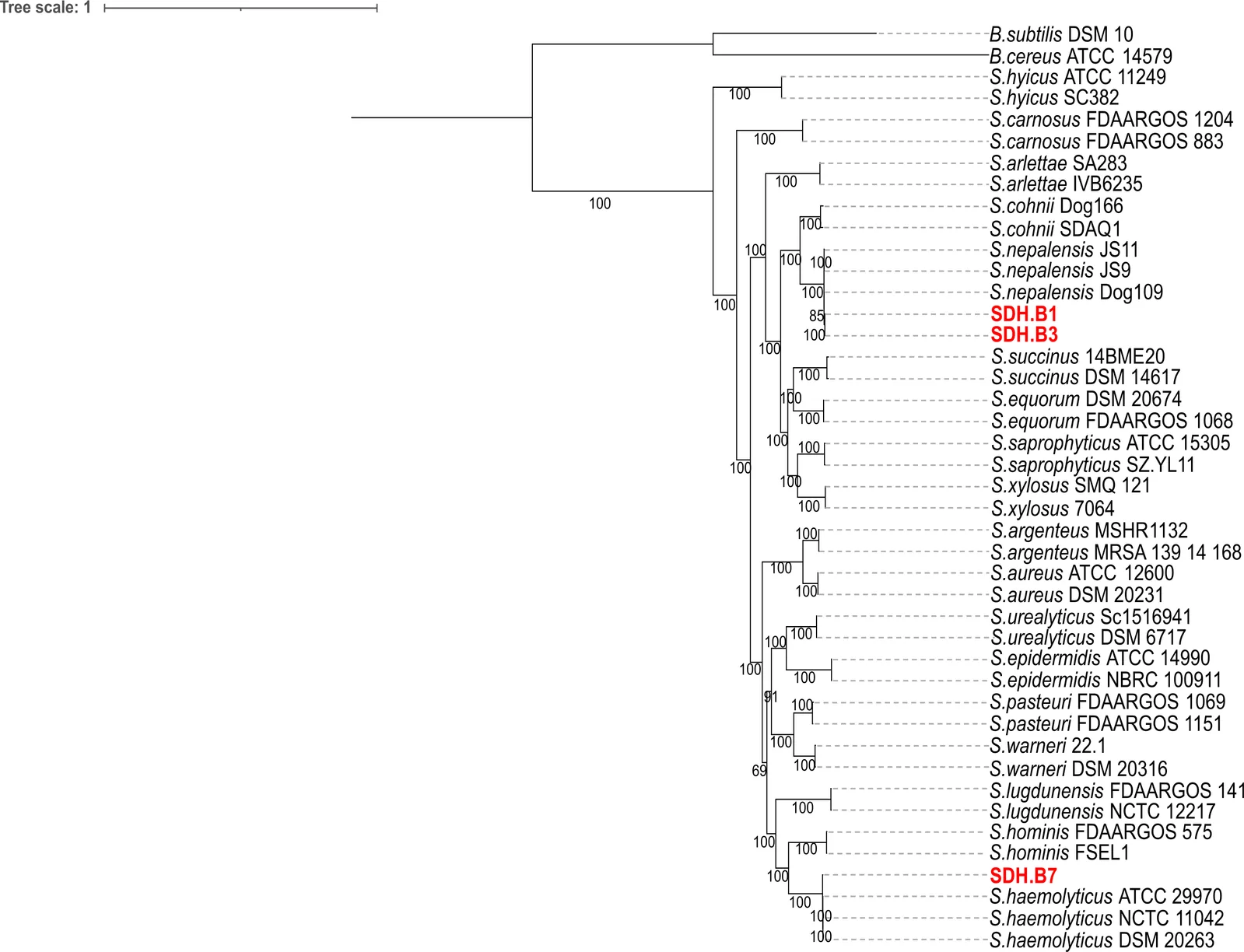
Core-gene phylogeny showing the relationships of SDH.B1, SDH.B3, and SDH.B7 to reference species within the genus *Staphylococcu*s. Two *Bacillus s*pecies were included as outgroups. The list of core genes used is listed in the Supplemental data.

A core-gene phylogeny including SDH.B1, SDH.B3, SDH.B7, 37 *Staphylococcus* reference species, and two *Bacillus* outgroups showed strong support for the placement of the study isolates (Fig. 4). SDH.B1 and SDH.B3 clustered with *Staphylococcus nepalensis*, whereas SDH.B7 clustered with *Staphylococcus haemolyticus*. These sequence-based assignments differed from the preliminary MALDI-TOF identifications for SDH.B1 and SDH.B3 but were strongly supported by the phylogenetic topology.

**Fig 5:**
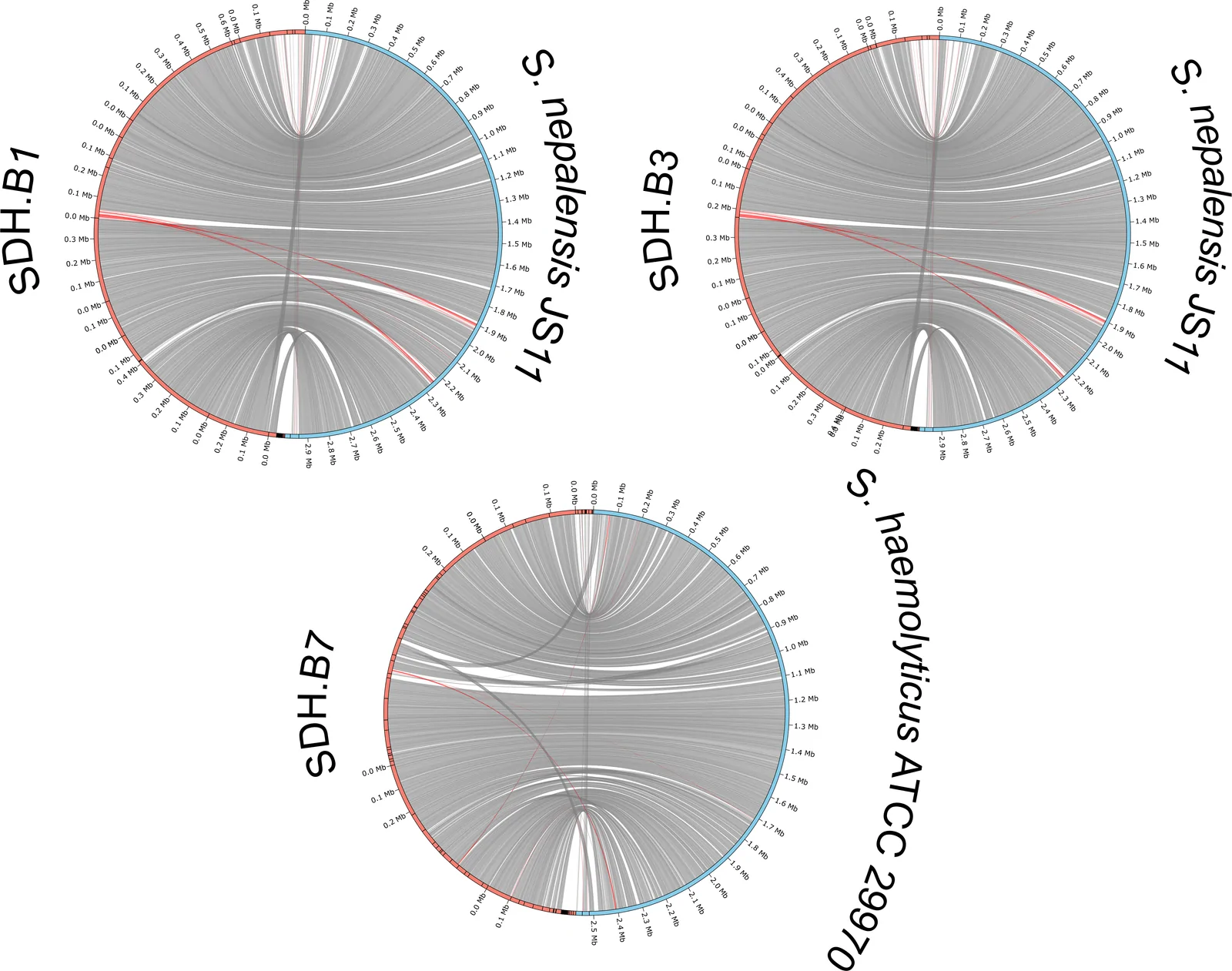
Circos plots showing genome alignment of SDH.B1, SDH.B3, and SDH.B7 against their respective reference genomes, *Staphylococcus nepalensis* JS11 and *Staphylococcus haemolyticus* ATCC 29970. Red chords indicate inversions, and gray chords indicate collinear regions.

A comparison with the relevant references (*S. haemolyticus* ATCC 29970 for B7; *S. nepalensis* JS11 for B1/B3) reveals distinct segments that reverse orientation (red chords) and broad collinearity (gray chords) (Fig. 5). B1 and B3 display compact, largely collinear maps with a small number of putative inversions; B7 shows more structural breaks, indicated by the more fragmented assembly, and might reflect unresolved repeat regions rather than genome rearrangement. These assessments were further supported by ANI. Notably, a direct pairwise comparison between SDH.B1 and SDH.B3 revealed an ANI of 99.99%, definitively confirming that they represent two isolates of a single *S. nepalensis* strain, with the 0.01% sequence difference might be attributable to minor technical sequencing noise. Furthermore, our data show that B1 and B3 share ~99.36%–99.40% ANI with *S. nepalensis* JS11 and that B7 shares ~99.21% with *S. haemolyticus* ATCC 29970. These results confirm that the isolates are closely related to known species, although subtle genomic differences remain when compared to reference strains.

### Multilocus sequence type profiles (MLST)

Among the three sequenced isolates, only SDH.B7 was assigned to sequence type 49 (ST49) using the available *S. haemolyticus* MLST scheme. No validated MLST scheme is currently available for *Staphylococcus nepalensis*; therefore, SDH.B1 and SDH.B3 could not be assigned to sequence types.

### Resistome analysis identified multiple antimicrobial and biocide resistance determinants

The three genomes carried determinants associated with resistance to antibiotics, antiseptics, heavy metals, and biofilm-related traits (Table 4). Tetracycline resistance in SDH.B1 and SDH.B7 was consistent with the presence of *tet(K*) and *tet(L*), both encoding MFS-type efflux pumps that actively extrude tetracycline from the cell (37). SDH.B3 carried *tet(K*) but remained phenotypically susceptible to tetracycline, highlighting a genotype–phenotype discordance that may reflect regulatory or expression-level effects not investigated here. This discordance is not without precedent in staphylococci—*tet*(*K*) expression is driven by a regulated promoter that can be silenced by upstream mutations, insertion elements, or phase variation, any of which could render the gene transcriptionally inactive despite its physical presence in the genome. Whether this reflects a stable loss of promoter function or a reversible regulatory state in SDH.B3 was not investigated here and would require expression-level analysis to resolve. Macrolide-lincosamide resistance was consistent across isolates with the presence of *erm*(B) in SDH.B1 and SDH.B3 and *erm*(C) in SDH.B7, both encoding 23S rRNA methyltransferases that prevent macrolide binding at the ribosome (38).

**TABLE 4:** Resistance genes and adhesion-associated loci detected in SDH.B1/B3 and SDH.B7, grouped by antibiotic resistance, biocide resistance, heavy metal resistance, and biofilm-associated categories

Gene	SDH.B1/B3	SDH.B7
Antibiotic resistance	*mecI*, *mecR1*, *mecA*, *blaZ*, *femA*, *femB*, *dfrE*, *aac(6*′*)-Ie/aph-(2*″*)*, *erm(B)*, *catA*, *fosB*, *tet(K)*, *arr*	*mecA*, *blaPC1*, *blaR1*, *blaI*, *femA*, *femB*, *erm(C)*, *tet(L)*, *aadD1*, *bleO*, *fusB*, *aac(6*′*)-Ie/aph-(2*″*)*, *mupA*
Biocide resistance	*qacG*, *sdrM*, *sepA*, *norB*	*qacA*, *qacR*, *sdrM*, *sepA*, *mepA*, *norA*
Heavy metal resistance	*arsB*, *arsC*, *cadD*	*zntA*, *arsB*, *arsC*, *arsD*, *cadC*, *cadD*
Biofilm forming	*veg*, *ebpS*, *eno*, *icaD*	*veg*, *ebpS*, *eno*

All three isolates carried *mecA*, which encodes the low-affinity penicillin-binding protein PBP2a and is the primary genetic basis for methicillin resistance in staphylococci. A notable difference, however, was that SDH.B1 and SDH.B3 also carried the regulatory genes *mecI* and *mecR1*, whereas SDH.B7 did not. In the classical mec regulatory circuit, MecI acts as a transcriptional repressor that suppresses *mecA* expression in the absence of β-lactam challenge, while MecR1 functions as a membrane-bound signal transducer that proteolytically inactivates *mecI* upon β-lactam exposure, thereby allowing *mecA* induction (39). The absence of *mecI* and *mecR1* in SDH.B7 means that *mecA* in this isolate is likely expressed constitutively, albeit at variable levels depending on genetic context. This regulatory difference may partly explain any subtle phenotypic variation in β-lactam resistance levels between the two species, although all three isolates were phenotypically methicillin-resistant, confirming that *mecA* is functional regardless of regulatory configuration. All three genomes also carried *blaZ*, encoding a β-lactamase that hydrolyzes narrow-spectrum penicillins. SDH.B7 additionally contained the complete *bla* regulatory operon—*blaI, blaR1*, and *blaPC1*—through which β-lactam-inducible expression of *blaZ* is coordinated (40, 41).

The co-occurrence of *mecA* and *blaZ* in all three isolates provides a layered genetic basis for the observed resistance to cefoxitin and oxacillin. All three isolates also carried *femA* and *femB*, which are required for assembly of the pentaglycine interpeptide bridge in staphylococcal peptidoglycan and are known to potentiate full expression of mecA-mediated resistance (42). The aminoglycoside resistance gene *aac(6*′*)-Ie/aph*(2″), encoding a bifunctional acetyltransferase–phosphotransferase enzyme, was identified in all three genomes. This determinant confers resistance to gentamicin, tobramycin, and related aminoglycosides through enzymatic modification of the drug. However, the observed aminoglycoside susceptibility profiles were not fully concordant with gene presence—a pattern that has been reported in CoNS and may reflect differences in enzyme expression levels, substrate specificity variation among aac(6′) variants, or the activity of compensatory mechanisms that restore partial susceptibility (43, 44).

Finally, SDH.B1 and SDH.B3 carried *catA* and *fosB*, which encode chloramphenicol acetyltransferase and a fosfomycin-resistance thiol transferase, respectively, while SDH.B7 carried *bleO* and *fusB*, associated with bleomycin sequestration and fusidic acid resistance. These isolates were not tested against the corresponding agents in this study; their detection nevertheless points to a broader resistance potential that warrants phenotypic confirmation in future work.

### Genomic localization and structural characterization of resistance determinants

Genomic localization of resistance determinants revealed a distinct spatial partitioning between chromosomally anchored elements and mobile plasmid replicons. Detailed genomic characterization via SCCmecFinder demonstrated that the primary driver of methicillin resistance, *mecA*, was chromosomally encoded across all three isolates, yet resided within fundamentally different cassette architectures. In SDH.B1/B3, *mecA* was located within a complete *mec* regulatory operon consisting of *mecI*, *mecR1*, and *mecA* (Class A *mec* complex); however, the surrounding cassette structures were not assignable to a recognized SCC*mec* type based on the present assembly. In contrast, in SDH.B7, *mecA* was positioned within a confirmed SCC*mec* Type V (5C2&5) element, characterized by a Class C2 *mec* complex coupled with the *ccrC1* allele 8 recombinase gene (Table 5).

**TABLE 5:** Genomic architecture of SCC*mec* elements and plasmid replicons in SDH.B1/B3 and SDH.B7

Isolates	Element	Location	Classification	Mobility and key markers	Carried resistance and genes
SDH.B1/B3	SCC*mec*	Chromosome	Untypeable	Chromosomally integrated	Class A *mec* complex: *mecI–mecR1–mecA*
	Plasmid AA367	Plasmid	Cluster 1078	Mobilizable (rep+, relaxase+)	*catA*
	Plasmid AC333	Plasmid	Cluster 1118	Mobilizable (rep+, relaxase+)	*tet(K*)
	Plasmid AB730	Plasmid	Cluster 1017	Non-mobilizable	*qacG*, *lnu(A)’*
SDH.B7	SCC*mec*	Chromosome	Type V (5C2&5)	Chromosomally integrated	Class C2 *mec* complex: *mecA*, *ccrC1* recombinase
	Plasmid AC670	Plasmid	Cluster 1018	Mobilizable (rep+, MOBv relaxase+)	*erm(C*), *tet(L)*, *aadD1*, *bleO*
	Plasmid AD194	Plasmid	Untypeable	Non-mobilizable	*mupA*, *qacA*, *qacR*
	Plasmid AA770	Plasmid	Untypeable	Non-mobilizable	*fusB*

Cross-referencing all detected resistance markers with MOB-suite plasmid predictions confirmed that the majority of core resistance determinants—including the entire bla operon (*blaI*, *blaR1*, *blaZ/blaPC1*), essential cell-wall synthesis factors (*femA*/*femB*), *dfrE*, *aac(6*′*)-Ie/aph(2*″), *erm(B*), *fosB*, *arr*, and all heavy metal resistance operons (*ars*, *cad*, *zntA*)—were chromosomally encoded in all three isolates. A distinct subset of resistance determinants, however, was plasmid-borne. In SDH.B1/B3, *catA* and *tet(K*) were each carried on separate plasmids (designated AA367 and AC333, respectively) that possessed both a rep gene and a relaxase gene and were accordingly classified as mobilizable; *qacG* and *lnu(A)*′ co-occurred on a third plasmid (AB730) lacking relaxase and *mpf* genes and were therefore classified as non-mobilizable, although its rep type is shared with sequences detected on multiple other contigs, suggesting broader distribution of this replicon family (Table 5). In SDH.B7, *erm(C*), *tet(L*), *aadD1*, and *bleO* co-occurred on a single mobilizable plasmid (AC670) bearing both a rep and a MOB_v_-family relaxase gene, while *mupA*, *qacA*, and *qacR* co-occurred on a second, considerably larger plasmid (AD194) for which no replication or mobilization gene could be identified despite its size, and *fusB* was carried on a further non-mobilizable plasmid (AA770). The structural co-existence of a chromosomally fixed β-lactam resistance backbone (mediated by SCC*mec* and bla operons) alongside dynamic, multi-resistance mobilizable plasmids indicates that horizontal gene transfer represents a major parallel pathway for multidrug resistance dissemination in CoNS, independent of chromosomal cassette evolution.

### Known disinfectant-associated efflux genes were identified in the representative isolates

In this study, all three isolates carried *qac* genes, consistent with the preliminary phenotypic screening and high biocide MIC values. SDH.B1 and SDH.B3 carried *qacG*, which belongs to the SMR family. SDH.B7 carried *qacA*, which encodes a major biocide efflux pump of the MFS family.

Comparison of the SDH.B7 QacA sequence with a reference QacA sequence showed high conservation, with only five amino acid substitutions (A152V, A184V, V188I, A291T, and M380T). Two substitutions, A291T and M380T, have previously been reported to have no apparent effect on QacA function (45). The remaining conservative changes are within predicted transmembrane regions and unlikely to abolish efflux activity, although this inference requires confirmation.

QacG is an SMR-family efflux determinant that has been associated with reduced susceptibility to quaternary ammonium compounds such as BKC (46). Its presence in SDH.B1 and SDH.B3 was consistent with the elevated BKC MICs observed in these isolates compared to the reference strain. Moreover, genome mining also identified six previously uncharacterized short membrane proteins, designated uSMR1 to uSMR6. uSMR1 to uSMR4 were detected in SDH.B1 and SDH.B3, whereas uSMR5 and uSMR6 were detected in SDH.B7.

**Fig 6:**
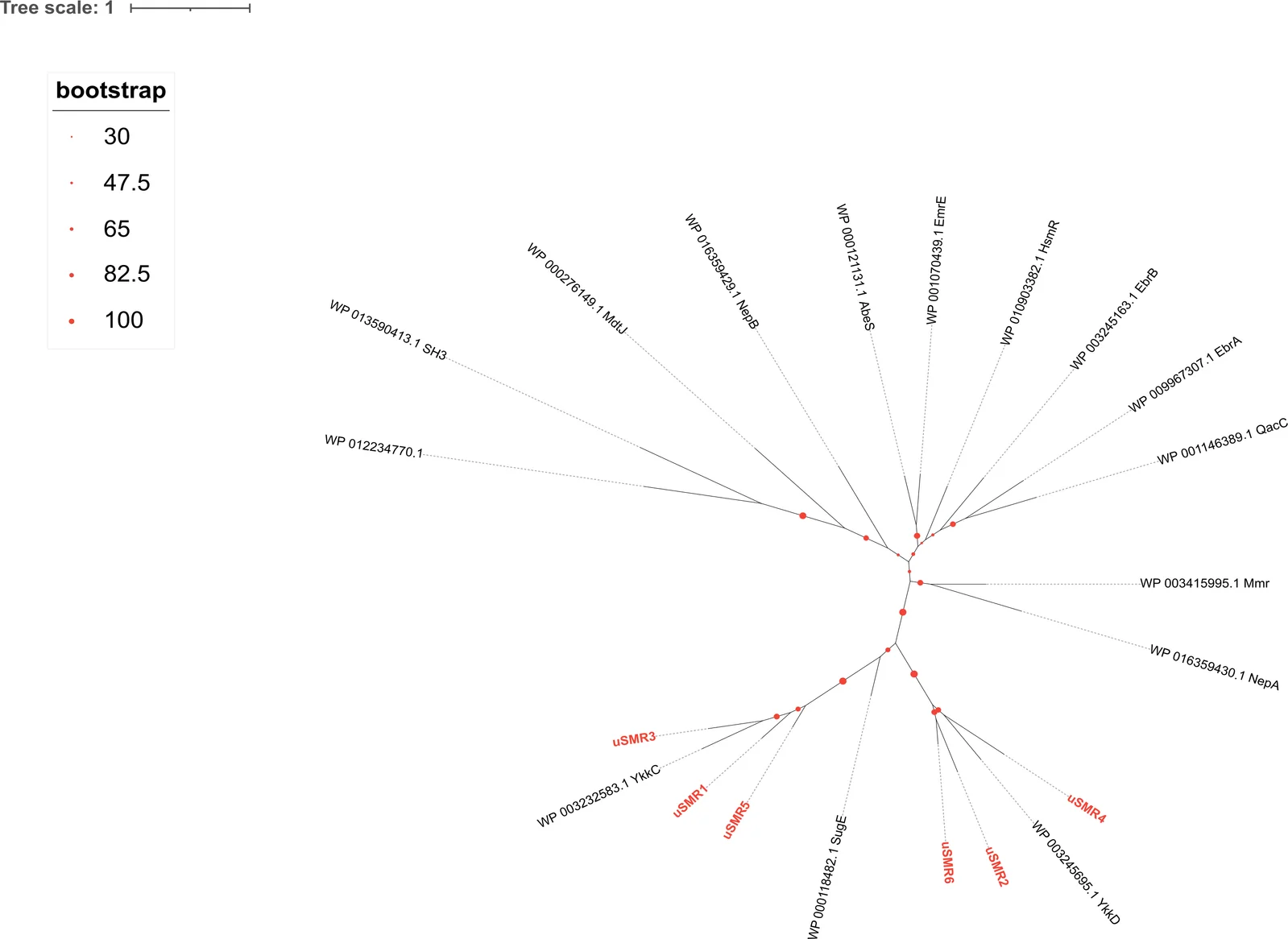
Phylogenetic tree of six putative SMR transporters (uSMR1–uSMR6) relative to 15 TCDB-curated SMR proteins. Amino acid sequences were aligned with MUSCLE v5, and a maximum-likelihood tree was inferred. Node support is shown by dot size.

Using the Transporter Classification Database (TCDB; http://www.tcdb.org) as a reference, we constructed a phylogenetic tree of 15 known SMR family proteins, including the six uSMRs (Fig. 6). The tree suggests that these new proteins cluster most closely with YkkC, YkkD, and SugE. The YkkC–YkkD pair has been reported in *Bacillus subtilis* and is thought to form a heterodimer that confers resistance to a wider range of toxic compounds than typical SMR family pumps (47). To understand their biochemical features, we analyzed these proteins using the ExPASy ProtParam server (Table 6). All six ranged from 103 to 109 amino acids—well within the expected size for SMR family transporters. Their instability index values were below 40. A protein is considered stable when its instability index value falls below 40, while a protein is classified as unstable if its instability index value exceeds 40 (48). The positive GRAVY scores suggest that they are quite hydrophobic and thus unlikely to interact favorably with water.

**TABLE 6:** Predicted physicochemical properties of six putative SMR transporters (uSMR1–uSMR6) computed with ExPASy ProtParam, including length, theoretical pI, molecular weight (Da), instability index, and grand average of hydropathy (GRAVY) scores

Protein	Number of amino acids	Theoretical pI	Molecular weight (Da)	Instability index	GRAVY
uSMR1	106	4.44	11,473.86	27.11	1.387
uSMR2	105	9.92	11,299.89	24.01	1.254
uSMR3	108	6.53	11,739.10	24.68	1.274
uSMR4	104	9.65	11,421.87	32.12	1.142
uSMR5	109	8.09	11,815.40	21.24	1.270
uSMR6	103	9.82	11,644.22	32.14	1.179

### Structural prediction and sequence analysis of uSMR complexes

**Fig 7:**
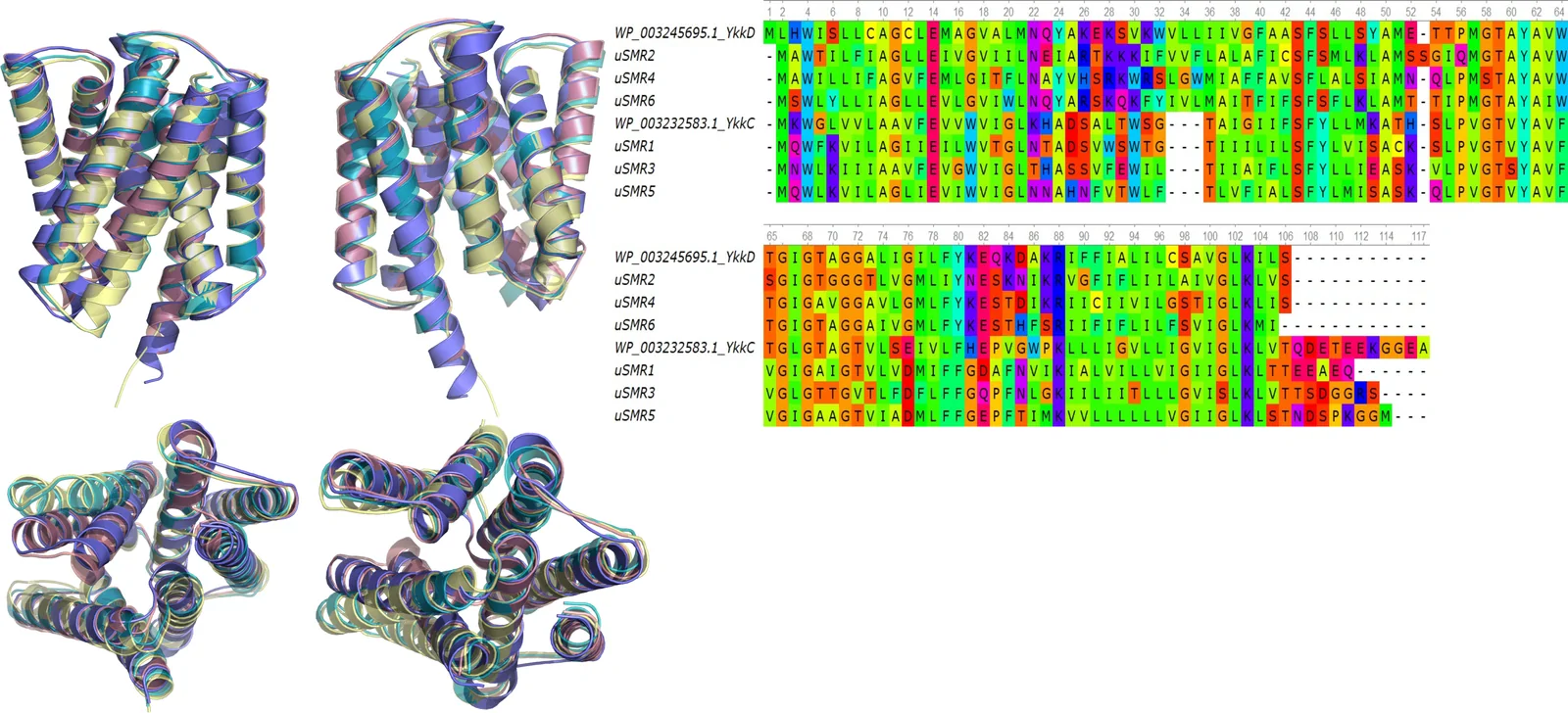
Structural models and sequence alignments of putative SMR-like proteins. Left, AlphaFold 2 models of uSMR pairs (uSMR1/uSMR2, teal; uSMR3/uSMR4, salmon; uSMR5/uSMR6, pale yellow) superposed on the YkkC/YkkD template (slate). Right, MUSCLE v5 multiple sequence alignment highlighting conserved residues among the putative SMR-like proteins and YkkC/YkkD-related sequences.

To evaluate the structural relationship between the hypothetical proteins and the YkkC–YkkD pair, we generated 3D heterodimeric models of uSMR1–uSMR2, uSMR3–uSMR4, and uSMR5–uSMR6 using AlphaFold 2 (AF2). In parallel, multiple sequence alignments were performed using MUSCLE v5 and visualized with Ugene (Fig. 7). AF2 predicted four transmembrane α-helices per monomer for all constructs. Sequence alignments revealed that the uSMRs share moderate overall amino acid identity with YkkC and YkkD. Notably, several conserved residues were identified across all uSMR and YkkC/YkkD sequences, localizing primarily within the transmembrane helices. These conserved positions are likely critical for maintaining the function of SMR family transporters; in particular, E13 has been previously implicated in interactions with cationic detergents (49).

To assess the reliability of these predicted models, we evaluated standard AF2 confidence metrics, including pLDDT (predicted Local Distance Difference Test), PAE (Predicted Aligned Error), ipTM (interface predicted Template Modeling), and pTM scores (Table 7). All four heterodimeric complexes demonstrated high confidence levels: average pLDDT values ranged from 88.0 to 92.8, categorizing them within the “Confident” to “Very high confidence” range. Furthermore, both pTM (0.84–0.89) and ipTM (0.81–0.87) scores consistently exceeded the 0.8 threshold, indicating highly reliable predictions for interchain interactions. The uSMR1/uSMR2 complex exhibited the highest overall reliability, whereas uSMR5/uSMR6 displayed the lowest within the uSMR group, although its scores remained comparable to the YkkC/YkkD reference (pLDDT: 88.4; ipTM: 0.81).

**TABLE 7:** AlphaFold 2 confidence metrics for the predicted uSMR heterodimer protein and the YkkC/YkkD reference^*a*^

Protein	Mean pLDDT	pTM	ipTM
uSMR1/uSMR2	92.8	0.89	0.87
uSMR3/uSMR4	90.4	0.87	0.85
uSMR5/uSMR6	88.0	0.84	0.82
YkkC/YkkD	88.4	0.85	0.81

^*a*^ pLDDT, predicted local distance difference test (per-residue confidence, averaged across the complex); pTM, predicted template modeling score; ipTM, interface predicted template modeling score (confidence in the predicted interchain interface).

To quantitatively evaluate structural similarity beyond visual superimposition, we calculated TM-scores and root-mean-square deviation (RMSD) for pairwise comparisons (Table 8). All three uSMR pairs exhibited high structural similarity to YkkC/YkkD, with TM-scores ranging from 0.87 to 0.96 and RMSD values between 0.77 and 2.23 Å (TM-scores > 0.5 indicate the same fold according to standard SCOP/CATH classifications) (50). Similarly, pairwise comparisons among the uSMR complexes themselves yielded high TM-scores (0.88–0.96) and low RMSD values (0.90–2.31 Å). When compared with the experimentally determined NMR structure of EmrE (51), the uSMR complexes also maintained high TM-scores of 0.83–0.84. Consequently, the AF2-predicted heterodimers adopt a highly conserved spatial architecture characterized by the SMR family transmembrane helices, closely mirroring the empirical EmrE structure.

**TABLE 8:** Pairwise sequence and structural comparison among YkkC/YkkD, the three uSMR protein pairs, and the experimentally determined reference transporter EmrE (PDB 8UWU)^*a*^

Protein	uSMR1/uSMR2	uSMR3/uSMR4	uSMR5/uSMR6	YkkC/YkkD	EmrE
uSMR1/uSMR2	–	0.90/2.03 Å	0.96/0.90 Å	0.88/1.93 Å	0.84/2.35 Å
uSMR3/uSMR4	48.1%	–	0.88/2.31 Å	0.96/0.77 Å	0.83/2.59 Å
uSMR5/uSMR6	59.3%	52.4%	–	0.87/2.23 Å	0.84/2.44 Å
YkkC/YkkD	48.6%	50.9%	52.4%	–	0.84/2.33 Å
EmrE	22.4%	25.2%	23.9%	24.3%	–

^*a*^ Values below the diagonal indicate pairwise amino acid sequence identity (%); values above the diagonal indicate structural similarity as TM-score/RMSD (Å) from pairwise structural alignment of AlphaFold 2 models. Diagonal cells (–) denote self-comparisons and are not applicable.

This high structural homology occurs in the context of moderate amino acid sequence identity both between the uSMR pairs and YkkC/YkkD (48.6%–52.4%) and among the uSMR pairs themselves (48.1%–59.3%). Meanwhile, identity with EmrE is markedly lower (22.4%–25.2%). This disparity suggests that the 3D architecture of the SMR family is subjected to significantly stricter evolutionary constraints than its primary sequence. The robust structural alignment with the experimental EmrE model confirms that this resemblance reflects genuine evolutionary conservation of the protein fold, rather than an artifact of comparing multiple AI-generated models against one another.

## DISCUSSION

This study demonstrates that disinfectant-tolerant CoNS are present on high-touch hospital surfaces in Vietnam and frequently display multidrug-resistant phenotypes. These findings support the view that environmental CoNS should not be considered passive contaminants only; rather, they may function as persistent reservoirs of resistance determinants within healthcare settings (52).

One of the most consistent findings of this study was the association between reduced disinfectant susceptibility and strong efflux-associated phenotypes. Baseline EtBr accumulation assays showed minimal intracellular dye retention in the hospital surface isolates compared with the reference strains. The addition of the EPI nicardipine successfully reversed this restricted retention. Quantitative AUC analysis confirmed a statistically significant increase in EtBr accumulation for 14 out of the 16 tested isolates upon EPI exposure. The magnitude of this EPI effect reached up to a 1.7-fold increase in specific isolates (e.g., SDH.B1), a pattern strongly indicative of efflux activity. This phenotypic observation was further supported by the presence of known disinfectant-associated efflux genes, including *qacA* in SDH.B7 and *qacG* in SDH.B1/B3. Although the present data do not directly quantify the expression of a broad spectrum of transporter families or substrate specificity, the EtBr accumulation assay still suggests that these efflux systems contribute to the reduced susceptibility observed in these hospital-derived isolates. These results align with previous studies showing that QacA and QacG are important contributors to quaternary ammonium resistance in staphylococci (53). Comparative analysis of the QacA sequence revealed only a few amino acid substitutions, most of which are unlikely to affect function, consistent with the observed resistance phenotype.

The coexistence of disinfectant tolerance and multidrug resistance is particularly concerning for infection control. All disinfectant-tolerant isolates were methicillin-resistant, yet the genomic architecture underlying this resistance differed. SDH.B1/B3 harbored *mecA* within a complete regulatory operon (Class A *mec* complex) embedded in an untypeable SCC*mec*, whereas SDH.B7 carried *mecA* within a confirmed SCC*mec* Type V element. This structural heterogeneity indicates that methicillin resistance in this collection is not the product of a single clonal expansion but rather reflects at least two independent evolutionary trajectories.

This broad resistome cannot be attributed solely to chromosomal fixation. Genomic localization revealed that while core resistance determinants are chromosomally anchored, several critical resistance genes reside on distinct plasmids. Notably, mobilizable plasmids actively contribute to multidrug resistance dissemination, as seen in SDH.B7, where *tet(L*), *erm(C*), *aadD1*, and *bleO* co-occurred on a single mobilizable replicon (AC670).

We also observed the physical linkage of biocide-tolerance and antibiotic-resistance determinants on the same replicons, such as *qacG* with *lnu(A)′* (plasmid AB730 in SDH.B1/B3) and *qacA/R* with *mupA* (plasmid AD194 in SDH.B7). Although these specific plasmids were classified as non-mobilizable or lacked identifiable mobilization machinery in our assembly, their co-localization provides a direct genomic mechanism by which disinfectant exposure could co-select for antibiotic resistance. Taken together, the structural co-existence of a chromosomally fixed resistance backbone alongside diverse multi-resistance plasmids highlights how plasmid-mediated co-selection drives the parallel spread of biocide and antimicrobial resistance in hospital-associated CoNS populations.

Alongside efflux pump diversity, biofilm formation was a prominent feature of multidrug-resistant isolates. Interestingly, we observed some phenotypic variation in biofilm-forming capacity and cell surface hydrophobicity between SDH.B1 and SDH.B3, two isolates confirmed to represent a single *S. nepalensis* strain (ANI = 99.99%). This may be related to their differing cell-surface hydrophobicity profiles (Fig. 3B). Initial bacterial adhesion to non-biological surfaces is a prerequisite for biofilm development, largely governed by hydrophobic interactions (54, 55). Our data showed that SDH.B3 maintained high CSH, while SDH.B1 exhibited significantly reduced hydrophobicity, illustrating that phenotypic expression can fluctuate *in vitro* even within clonal isolates.

The presence of biofilm-related genes in the WGS data did not always correlate with phenotypic expression. Such genotype–phenotype differences have been clearly observed in staphylococci (56). The variation in surface hydrophobicity between these two clonal isolates suggests the transient absence, masking, or differential expression of crucial hydrophobic surface-anchoring proteins. The loss of surface hydrophobicity in SDH.B1, despite its nearly identical genome to SDH.B3, suggests the absence or masking of crucial hydrophobic surface-anchoring proteins. In closely related CoNS species, large surface proteins (e.g., SxsA or Sdr family proteins) determine these hydrophobic interactions (57). Therefore, this phenotype is likely driven by regulatory silencing, transcriptomic plasticity, or phase-shifted structural modifications, such as truncation of parallel repeat domains in surface proteins. Because our genomic analysis relied on standard short-read whole-genome sequencing, which often collapses highly repetitive regions and cannot capture epigenetic or transcriptomic variations, we are currently unable to pinpoint the exact mechanisms responsible for this intra-strain phenotypic variation. This may result in SDH.B1 failing at the crucial initial adhesion step due to impaired hydrophobicity; however, further experiments utilizing long-read sequencing or RNA-seq are needed to confirm these mechanisms.

Recognizing biofilm’s role in resistance can reinforce the importance of infection control practices among healthcare professionals, as the coexistence of efflux-mediated biocide tolerance and robust biofilm formation may provide a dual advantage, protecting the bacteria at both the cellular and community levels. Recent studies confirm that efflux pumps not only extrude antimicrobial compounds but also modulate quorum sensing and biofilm architecture, contributing to multidrug tolerance (58). Understanding these combined mechanisms highlights the relevance of ongoing research and can motivate efforts to develop more effective infection control strategies in clinical settings.

A major contribution of this work is the identification and structural characterization of six previously uncharacterized short membrane proteins, designated uSMR1 through uSMR6. While homologous sequences are present in public databases for *S. nepalensis* JS11 and *S. haemolyticus* ATCC 29970 under generic “Drug/Metabolite Transporter (DMT) family” annotations (Supplemental data), their functional annotation and structural interpretation as SMR-like proteins have remained unclear. By resolving their 3D architectures, our findings provide structure-based annotation of the SMR family. Their size (103–109 amino acids), positive GRAVY scores, four transmembrane helix topology, and phylogenetic clustering near the established YkkC/YkkD system collectively support their reclassification as a distinct SMR-like group in CoNS.

To rigorously validate these structural hypotheses, we benchmarked the AI-generated heterodimers against EmrE, a paradigmatic and experimentally resolved SMR efflux pump from *Escherichia coli*. Despite sharing a minimal primary sequence identity of approximately 22%–25% with EmrE, the uSMR complexes exhibited high structural homology, yielding TM-scores well above the threshold for shared fold classification. The decoupling of primary sequence divergence from 3D structural conservation provides a compelling evolutionary insight. It strongly suggests that these newly identified proteins possess functional capabilities and biological interaction dynamics analogous to well-characterized SMR efflux pumps. Furthermore, this structural rigidity in the face of sequence divergence points toward strong purifying selection acting on the SMR fold: even as sequence identity has drifted with evolutionary distance, the selective pressures imposed by ubiquitous biocides and detergents appear to constrain the transmembrane architecture to a narrow structural solution for toxic-compound extrusion, consistent with the shared ancestry of uSMRs with YkkC/YkkD/SugE established in our phylogenetic analysis.

Nevertheless, it is important to acknowledge that the evidence supporting the functional assignment of the uSMRs is currently computational. While the high-confidence metrics of our predictive models (pLDDT, ipTM, TM-scores) strongly substantiate their biological plausibility, actual transport activity, specific substrate spectra, and *in vivo* oligomeric states have yet to be empirically verified. Therefore, these proteins are currently best defined as putative SMR-like transporters.

To move beyond this computational baseline, future studies should prioritize functional validation through well-established experimental approaches. Heterologous expression of individual uSMR genes—or co-expression of pairs presumed to act as heterodimers—in a suitable host such as *E. coli* or a *qac*-null *S. aureus* background would allow direct assessment of whether these proteins confer reduced susceptibility to quaternary ammonium compounds or other disinfectants. EtBr accumulation assays with strains overexpressing individual uSMR genes would provide a straightforward first readout of efflux activity. Conversely, targeted gene deletion or knockdown in the native isolates, followed by re-measurement of BKC or CHG MICs, would test whether any of these proteins contribute meaningfully to the tolerance phenotype already documented here. Taken together, such experiments would clarify whether the uSMRs are active transporters, structural paralogs with diverged function, or genomic remnants—and in doing so, determine whether they represent a genuinely new class of efflux determinants in hospital-associated CoNS.

From a broader perspective, the coexistence of efflux-associated traits, disinfectant tolerance, multidrug resistance, and biofilm formation may provide environmental CoNS with a substantial survival advantage in hospital ecosystems. Such combinations of traits could facilitate persistence on abiotic surfaces, reduce the effectiveness of routine decontamination procedures, and increase the likelihood that resistant strains remain available for onward transmission. These findings reinforce the need to include environmental CoNS in surveillance programs aimed at understanding how resistance determinants circulate within healthcare environments.

This study has several limitations. First, the number of sequenced isolates was limited, and all samples were obtained from a single hospital, which constrains the generalizability of the findings. Second, the roles of the newly identified uSMR proteins were inferred from sequence and structural analyses rather than confirmed experimentally. Third, gene content did not always translate directly into phenotype, indicating that additional regulatory or physiological factors are likely involved. Future work should therefore include broader environmental sampling, expression analyses, and functional assays such as gene knockout or heterologous expression to determine the contribution of individual efflux systems to disinfectant tolerance.

## Data Availability

Draft genome assemblies for SDH.B1, SDH.B3, and SDH.B7 have been deposited in GenBank under accession numbers JBPUTG000000000, JBPUTH000000000, and JBPUTI000000000, respectively.

## SUPPLEMENTAL MATERIAL

Supplemental figure and tables spectrum.01294-26-s0001.pdf Fig. S1 and Tables S1 to S3.

ASM does not own the copyrights to Supplemental Material that may be linked to, or accessed through, an article. The authors have granted ASM a non-exclusive, world-wide license to publish the Supplemental Material files. Please contact the corresponding author directly for reuse.
