# Supplementary material for: Genomic characterization of efflux pumps and putative small multidrug resistance (SMR)-like transporters in disinfectant-tolerant coagulase-negative staphylococci from a Vietnamese hospital
Source: Microbiol Spectr. 2026 Sep 9;14(10):e01294-26. doi: 10.1128/spectrum.01294-26 (PMC13688860; doi:10.1128/spectrum.01294-26)

Table S1: Effect of nicardipine on intracellular ethidium bromide (EtBr) accumulation across 16 *Staphylococcus* strains. Fold change was calculated as AUC in the presence of nicardipine divided by AUC in the absence of nicardipine.

**Strain**	**Fold change**
SDH.B1	1.7
SDH.B2	1.6
SDH.B3	1.5
SDH.B51	1.4
SDH.B10	1.4
SDH.B7	1.4
SDH.B5	1.3
SDH.B8	1.3
SDH.B15	1.3
SDH.B39	1.3
SDH.B36	1.2
SDH.B32	1.2
SDH.B30	1.2
SDH.B34	1.2
S. aureus ATCC 25923	1.2
S. epidermidis ATCC 12228	1.2

Table S2: List of 38 housekeeping genes used in phylogeny showing the relationships of SDH.B1, SDH.B3, and SDH.B7 to reference species within the genus *Staphylococcus*.

No.	Gene	No.	Gene	No.	Gene	No.	Gene
1	16S rRNA	11	*ftsY*	21	*pheS*	31	*rpsF*
2	23S rRNA	12	*gmk*	22	*pyrH*	32	*rpsG*
3	*alaS*	13	*hisS*	23	*rplA*	33	*rpsL*
4	*clpP*	14	*ileS*	24	*rplS*	34	*rpsP*
5	*coaE*	15	*infB*	25	*rpmB*	35	*spxA*
6	*dnaA*	16	*infC*	26	*rpoB*	36	*tsf*
7	*dnaG*	17	*lepA*	27	*rpoC*	37	*udk*
8	*ffh*	18	*leuS*	28	*rpoD*	38	*ybeY*
9	*fmt*	19	*lipA*	29	*rpsB*		
10	*frr*	20	*nusA*	30	*rpsD*		

Table S3: tBLASTn top hits for the six uSMR proteins against the NCBI RefSeq Reference genomes database of *S. nepalensis* & *S. haemolyticus*.

Query Protein	Description	Accession Number	Query Cover	Per. Ident	E-value	Locus tag
**uSMR1**	DMT family transporter	NZ_CP017466.1	100%	78.30%	5.0×10^{-47}	BJG89_RS03775
**uSMR2**			99%	84.09%	3.0×10^{-41}	
**uSMR3**			100%	87.04%	6.0×10^{-52}	BJG89_RS13355
**uSMR4**			100%	100%	1.0×10^{-63}	
**uSMR5**		NZ_CP035291.1	100%	84.40%	7.0×10^{-54}	EQ029_RS03240
**uSMR6**			100%	68.93%	8.0×10^{-37}	EQ029_RS03235

Query Cover, percentage of the query sequence aligned to the hit; Per. Ident, percentage amino acid identity over the aligned region; E-value, expectation value of the alignment.

Figure S1: Structural predictions and confidence metrics for putative heterodimeric complexes. The figure presents the computationally predicted 3D models for four protein pairs: uSMR1/uSMR2, uSMR3/uSMR4, uSMR5/uSMR6, and YkkC/YkkD.

uSMR1(A)/uSMR2(B):

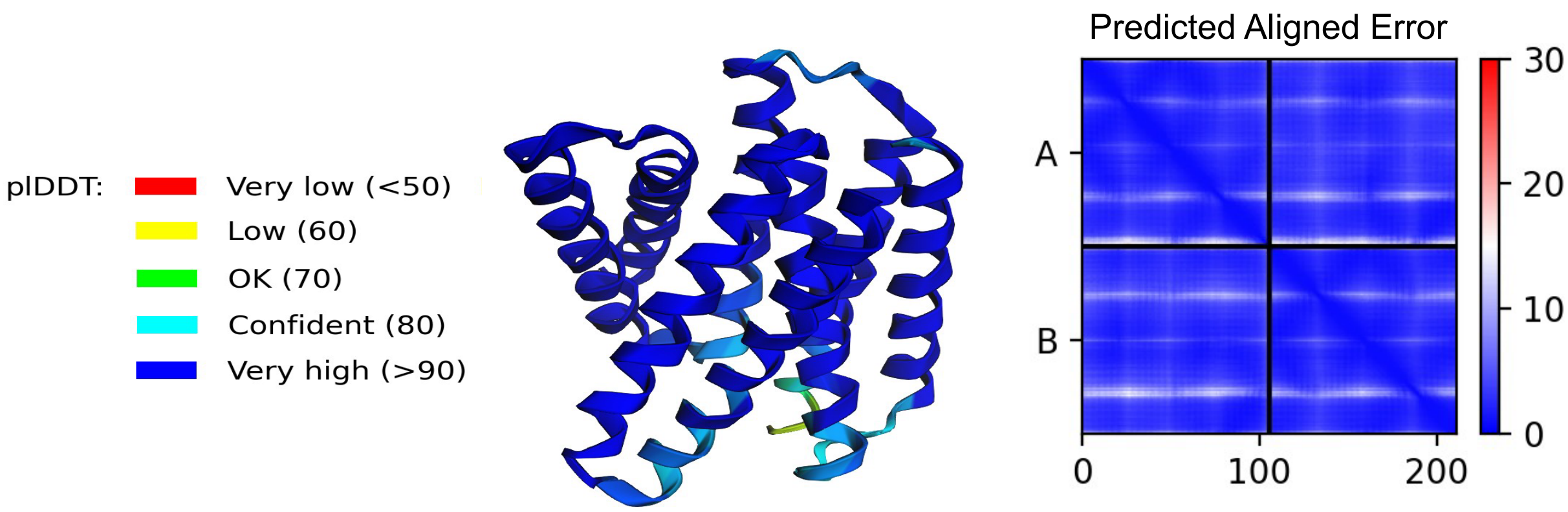

uSMR3(A)/uSMR4(B):

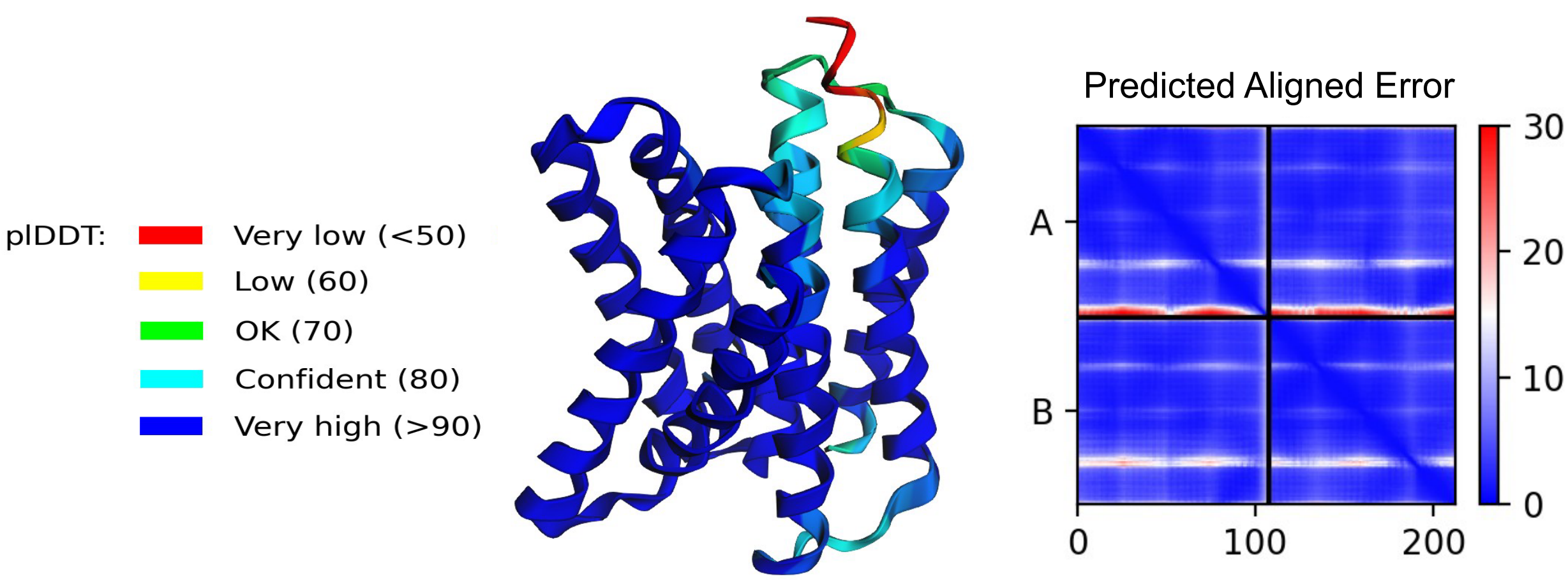

uSMR5(A)/uSMR6(B):

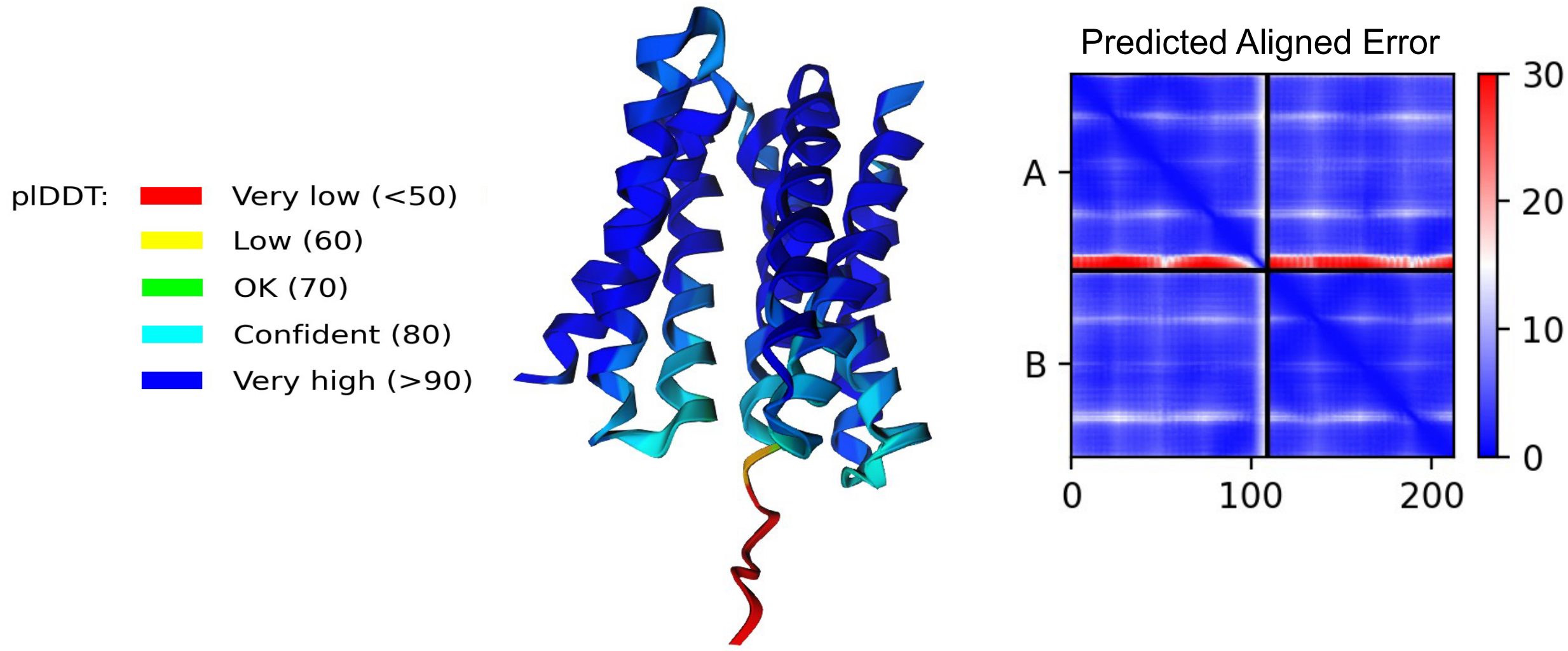

YkkC(A)/YkkD(B):

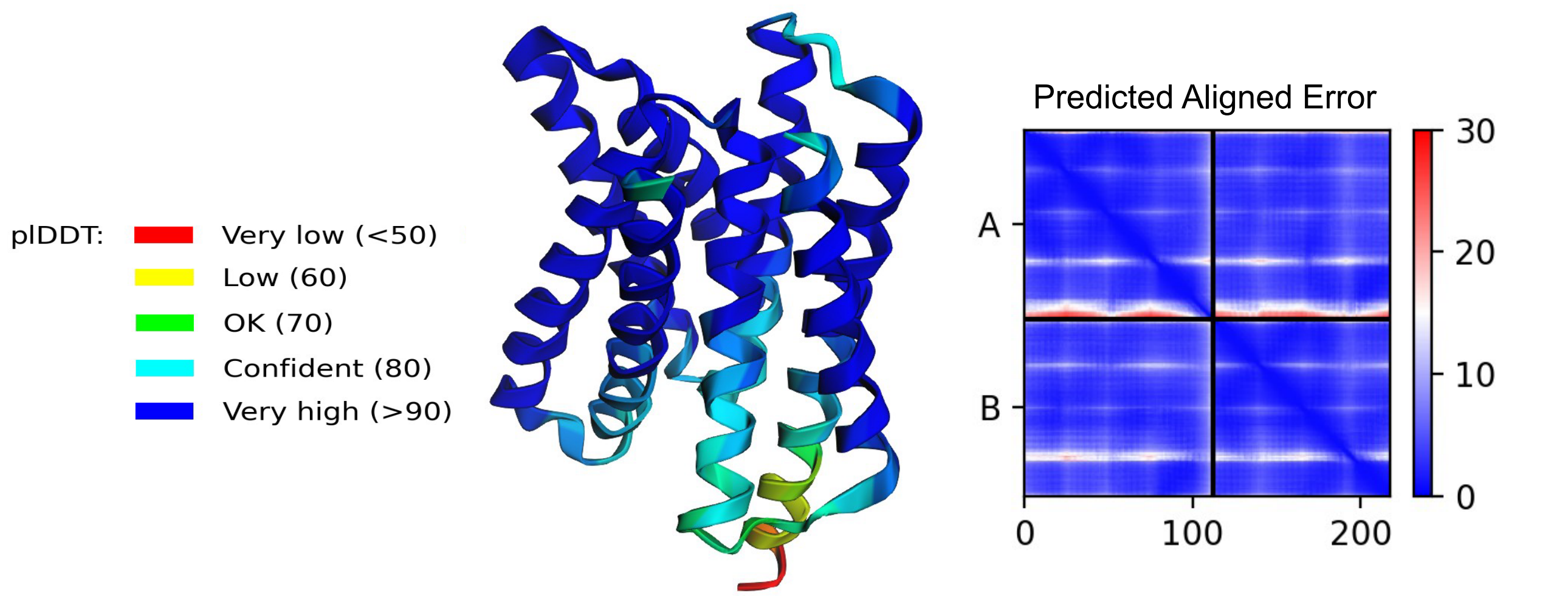

Supplement: Supplementary file 1 [file spectrum.01294-26-s0001.pdf]
